# Facility readiness and determinants of malaria test-before-treat implementation in public health facilities in Kaduna and Kano States, Nigeria

**DOI:** 10.1186/s12936-026-05949-2

**Published:** 2026-06-19

**Authors:** Uchenna Igbokwe, Zaharaddeen Babandi, Precious Uahomo, Yayo Abdulsalami Manu, Babangida Musa, Sule Danga, Hamza Muhammad, Fatima Mohammed Musa, Zainab Kwaru Muhammad-Idris, Idowu Akanmu, Shukrat Yusuff-Lawal, Osezefe Ehimen, Miftahu Yahaya, Nchelem Ichegbo, Olalekan Ojosipe, Joseph Natsah, Reuben Kaha, Tisan Gugong, Saheed Popoola, Oluwaseun Fadeyi, Eric Aigbogun

**Affiliations:** 1 Solina Centre for International Development and Research (SCIDaR), 8 Libreville Street, Off Aminu Kano Crescent, Wuse II, Abuja, FCT Nigeria; 2 Centre for Infectious Diseases Research (CIDR), Bayero University, Kano, Kano State Nigeria; 3 Centre for Mosquito-Borne Diseases Research and Community Health (CEMCOH), Kaduna State University, Kaduna, Kaduna State Nigeria; 4 State Malaria Elimination Program (SMEP), Kano, Kano State Nigeria; 5 State Malaria Elimination Program (SMEP), Kaduna, Kaduna State Nigeria

**Keywords:** Public health facility, malaria, test-before-treat, determinants, rapid diagnostic tests, readiness, subnational tailoring (SNT), Kano state, Kaduna state

## Abstract

**Background:**

Prompt diagnosis and treatment of malaria remain central to malaria control strategies. The World Health Organization recommends testing suspected malaria cases before treatment using rapid diagnostic tests (RDTs) or microscopy. However, evidence on the readiness of health facilities to implement this policy in high-burden Nigerian states remains limited. This study assessed facility readiness, testing-before-treatment (TBT) coverage, and facility-level determinants of malaria diagnostic readiness among public health facilities in Kaduna and Kano States, Nigeria.

**Methods:**

A cross-sectional health facility assessment was conducted across 418 public health facilities in Kaduna and Kano States between August 5 and 28, 2025. A stratified two-stage sampling approach was used, with primary health facilities randomly selected proportionally across all 67 Local Government Areas and all secondary facilities purposively included. Data were collected using a structured Health Facility Assessment questionnaire. Facility readiness was assessed using five indicators: availability of RDTs, availability of microscopy services, trained staff on malaria case management, availability of standard operating procedures, and absence of stock-outs of RDTs or artemisinin-based combination therapy within the previous three months. A composite readiness index (0–5) was constructed and facilities were classified as having full or partial readiness. Associations between facility characteristics and readiness were examined using multivariable logistic regression.

**Results:**

All facilities had RDTs and trained staff on malaria case management, and none reported commodity stock-outs during the three-month reference period. Overall, 381 facilities (91.1%) demonstrated full readiness, with a mean readiness score of 4.91 ± 0.29. Microscopy services were available in 23.0% of facilities. Most facilities reported high TBT coverage, with 90.4% reporting ≥ 85% testing coverage. Secondary facilities (aOR = 2.15, 95% CI:1.06–4.36), urban location (aOR = 1.78, 95% CI:1.01–3.12), supportive supervision (aOR = 2.54, 95% CI:1.28–5.02), malaria focal persons (aOR = 2.92, 95% CI:1.63–5.23), and availability of free malaria commodities (aOR = 1.98, 95% CI:1.07–3.68) were significantly associated with full readiness.

**Conclusion:**

Public health facilities assessed in Kaduna and Kano States demonstrated high structural readiness for malaria diagnosis. Study limitations include the cross-sectional design, reliance on facility records which may be subject to reporting bias, and the exclusion of private health facilities, limiting generalizability. These findings highlight the need for sustained commodity security, regular supportive supervision, and targeted investments in rural facilities to maintain and strengthen diagnostic readiness.

## Introduction

Malaria caused by *Plasmodium falciparum* remains a major public health challenge in sub-Saharan Africa, accounting for over 580,000 deaths annually and representing approximately 90% of global malaria deaths [1]. Nigeria bears a disproportionate share of this burden, accounting for approximately one-quarter of global malaria cases and deaths, with the greatest impact observed among children under five years of age and pregnant women [1]. According to the 2024 World Malaria Report, Nigeria contributes 25.9% of global malaria cases and 30.9% of malaria-related deaths, and accounts for approximately 55% of malaria cases in West Africa [1]. Access to prompt diagnosis and effective treatment with Artemisinin-based Combination Therapy (ACT) remains critical for reducing malaria morbidity and mortality [2, 3].

The World Health Organization (WHO) recommends that all suspected malaria cases be parasitologically confirmed using microscopy or malaria rapid diagnostic tests (RDTs) before treatment is initiated [3]. This recommendation is based on evidence that presumptive treatment of febrile illness as malaria may lead to missed alternative diagnoses and unnecessary treatment of patients who do not have malaria, potentially exposing patients to inappropriate medications, increasing healthcare costs, and contributing to the development of antimalarial drug resistance [4–6]. Nigeria has adopted the WHO “Test, Treat and Track” policy, which requires that all suspected malaria cases be tested using malaria RDTs or microscopy and that confirmed uncomplicated cases be promptly treated with effective ACTs [7]. In line with this policy, the National Malaria Strategic Plan (2021–2025) aims to ensure that at least 80% of suspected malaria cases receive appropriate diagnostic testing and treatment by 2025 [8].

Despite these policy commitments, presumptive malaria treatment continues to pose a challenge in Nigeria and may undermine progress toward malaria elimination. Testing-before-treatment (TBT) provides significant clinical and public health benefits by improving diagnostic accuracy, reducing unnecessary drug use, and enabling better management of non-malarial febrile illnesses [9–11]. However, evidence suggests that many patients with suspected malaria do not undergo diagnostic testing prior to treatment [10, 11]. This gap between policy and practice may be driven by several factors, including inadequate diagnostic capacity, stock-outs of malaria commodities, high patient volumes, insufficient training of healthcare workers, and weak adherence to national malaria treatment guidelines within health facilities [12].

Population-based surveys in Nigeria continue to demonstrate low diagnostic testing coverage among febrile children. The 2021 Nigeria Malaria Indicator Survey reported that only 24% of children with fever had blood taken for malaria diagnosis [13]. Similarly, studies conducted in different parts of Nigeria highlight persistent challenges in adherence to malaria diagnostic guidelines. In Ogun State, presumptive malaria treatment was reported in 94.9% of private health facilities and 22.7% of public facilities [14]. A study in Ebonyi State reported that approximately 20% of primary health workers still practiced presumptive malaria treatment [15]. In Rivers State, evidence suggests that key components of the national “test before treat” strategy, including testing all suspected malaria cases before treatment, have not been consistently implemented across health facilities [16]. Additionally, the WHO malaria report for Nigeria indicated that the proportion of febrile children who sought treatment and received malaria diagnostic testing was only 22% in Kaduna State and 30.6% in Kano State [17]. Even in settings where testing before treatment appears to be widely practiced, studies suggest that health workers may still treat patients for malaria despite negative test results due to clinical suspicion or lack of confidence in diagnostic tools [12]. Furthermore, reported high testing coverage in facility records may sometimes reflect data quality challenges, including incomplete documentation or misreporting of diagnostic practices [18].

Assessment of health facility readiness to provide malaria diagnostic services is essential for understanding the extent to which health systems are prepared to implement malaria control policies. Health facility readiness is commonly evaluated using structured frameworks such as the World Health Organization Service Availability and Readiness Assessment (SARA) framework, which assesses the availability of essential medicines, diagnostic tools, trained personnel, and clinical guidelines required to deliver health services effectively [19]. Similarly, national malaria programmes often employ service readiness indicators to monitor the capacity of health facilities to implement malaria diagnostic and treatment policies. These frameworks provide a systematic approach for evaluating whether health facilities possess the structural and operational requirements needed to support effective malaria case management.

Although earlier Nigerian studies have examined malaria diagnostic practices or adherence to treatment guidelines, most have been limited to single states, small samples, or private sector settings, and few have employed a composite readiness index aligned with national frameworks to assess diagnostic capacity across multiple states. Furthermore, the role of programmatic health system factors, such as supportive supervision, the presence of malaria focal persons, and commodity security, in influencing facility readiness remains underexplored. This study fills these gaps by providing a large-scale, multi-state assessment of facility readiness and testing-before-treatment coverage in 418 public health facilities across Kaduna and Kano States, two of Nigeria’s highest-burden settings. The findings offer actionable evidence for policymakers and malaria programme managers on how to strengthen diagnostic systems and sustain progress toward elimination targets. Therefore, this study aims to assess the level of malaria diagnostic readiness, the reported coverage of testing before treatment, and the facility-level factors associated with readiness for implementing malaria test-before-treat practices in public health facilities in Kaduna and Kano States, Nigeria.

## Methods

### Study design

A facility-based descriptive cross-sectional study was conducted to assess malaria diagnostic readiness and testing before treatment practices in public health facilities in Kaduna and Kano States, Nigeria. The cross-sectional design allowed for the systematic assessment of facility readiness indicators and malaria diagnostic practices at a single point in time across multiple health facilities.

### Study setting

The study was conducted in Kano and Kaduna States, both in Northwestern Nigeria. Kaduna State had a population of 6,113,503 as at the 2006 census, with a 2025 projected population of 9,190,000 in 23 Local Government Areas (LGAs) [20]. The State has a total of 1,068 Primary Healthcare facilities and 32 Secondary health facilities [21]. Kaduna State experiences two distinct seasons: the dry and wet seasons, with annual rainfall of about 1,175 mm. With a prevalence of 16.2% the State contributes an estimated 5.3% of Nigeria’s Malaria cases [17]. Kano State has a projected population of 16.2 million people across 44 LGAs. Kano State has a total of 1,142 Primary Healthcare facilities and 39 Secondary Healthcare facilities [22, 23]. The two states experience two distinct seasons: the dry season with annual rainfall averaging around 813.5 mm. With a prevalence of 25.5% the State contributes an estimated 9% of Nigeria’s malaria cases according to the WHO report on malaria in Nigeria [17]. The study population included the Primary Health Care and Secondary Health facilities in Kano and Kaduna States. This assessment was carried out between August 5th and 28th, 2025. Health facilities were classified as urban or rural based on the official administrative designation of their LGAs as defined by the National Population Commission and state-level administrative records. Facilities located within LGAs designated as metropolitan, municipal, or urban districts were classified as urban, while those located in non-metropolitan LGAs characterized by lower population density and predominantly rural settlement patterns were classified as rural.

### Conceptual framework for facility readiness

The assessment of malaria diagnostic readiness in this study was guided by principles from the WHO SARA framework, which is widely used to evaluate the capacity of health facilities to deliver essential health services. The WHO SARA framework conceptualizes service readiness as the availability of major inputs required for effective service delivery, including trained health personnel, diagnostic equipment, essential medicines, and clinical guidelines [19]. These components represent key structural elements of health systems that enable the implementation of clinical policies and treatment protocols.

Within malaria control programmes, health facility readiness is particularly important for the implementation of the “Test, Treat and Track” strategy, which requires that all suspected malaria cases be confirmed through parasitological testing before treatment. Successful implementation of this policy depends on the availability of diagnostic tools such as RDTs or microscopy, trained health workers capable of performing and interpreting tests, treatment commodities such as ACTs, and adherence to standard clinical guidelines [24].

In Nigeria, the National Malaria Elimination Programme (NMEP) has adopted service readiness indicators aligned with WHO recommendations to monitor the capacity of health facilities to implement malaria case management policies. These indicators focus on both structural and operational components of service delivery, including the availability of malaria diagnostic tools, trained personnel, standard operating procedures (SOPs) or clinical guidelines, and consistent availability of malaria commodities. Facility-level management factors, such as supportive supervision and the presence of designated malaria focal persons, are also recognized as important programmatic elements that strengthen adherence to malaria treatment guidelines and improve service delivery quality.

Drawing on these frameworks, this study operationalized facility readiness using five key indicators that reflect the minimum requirements for implementing the malaria test-before-treat policy at health facility level:

1. Availability of malaria RDTs
2. Availability of functional microscopy services
3. Presence of trained staff on malaria case management
4. Availability of malaria case management standard operating procedures
5. Absence of stock-outs of malaria commodities (RDTs or ACTs)

These indicators collectively represent both the structural readiness of health facilities (availability of commodities and diagnostic tools) and organizational readiness (trained personnel and clinical guidelines) necessary to support malaria diagnostic services. The framework assumes that when these readiness components are present, health facilities are better equipped to implement diagnostic testing prior to treatment and adhere to national malaria case management guidelines.

In addition to these readiness indicators, this study examined selected facility-level contextual factors that may influence readiness and implementation of diagnostic practices. These included facility type, geographic location (urban or rural), supportive supervision, availability of malaria focal persons, and programmatic support mechanisms such as Global Fund and Basic Health Care Provision Fund (BHCPF) support. These contextual factors reflect broader health system inputs that may facilitate or constrain the effective implementation of malaria diagnostic policies at facility level.

### Sample size determination

The unit of assessment in this study was the health facility. The sampling frame consisted of the official registry of public health facilities providing malaria diagnostic and treatment services obtained from the Kaduna and Kano State Ministries of Health and the State Primary Health Care Development Boards. At the time of the assessment, Kaduna State had approximately 1,068 primary health care facilities and 32 secondary facilities, while Kano State had approximately 1,142 primary health care facilities and 39 secondary facilities [21–23]. All secondary facilities in both states were purposively included; however, facilities that were non-functional, did not provide malaria diagnostic services, or had no staff available at the time of the survey were excluded (see inclusion and exclusion criteria). To ensure geographic representation, the sampling covered all 67 Local Government Areas (44 in Kano and 23 in Kaduna). Primary health care facilities were randomly selected within each LGA in proportion to the number of facilities in that LGA, while all secondary health facilities were purposively included. Using this approach, a total of 418 public health facilities were included in the assessment, comprising 177 facilities in Kaduna State and 241 facilities in Kano State. The target sample size of 418 facilities was determined to provide a precision of ± 5% around an expected proportion of 90% for key readiness indicators, assuming a design effect of 1.2 to account for modest clustering at the LGA level, at a 95% confidence level. All secondary health facilities were purposively included, and primary health centres were then allocated across LGAs in proportion to the number of facilities in each LGA, rounded up to the nearest integer, to achieve the overall target while maintaining geographic representativeness. The final number of 418 facilities (177 in Kaduna, 241 in Kano) slightly exceeded the minimum required sample of approximately 380 facilities.

### Inclusion and exclusion criteria

Health facilities were eligible for inclusion if they were public primary or secondary health facilities listed in the official registry of the Kaduna and Kano State Ministries of Health or State Primary Health Care Development Boards, provided malaria diagnostic and treatment services, and were operational with responsible staff available at the time of the assessment; facilities were excluded if they were private facilities, did not provide malaria services, were non-functional at the time of the survey, or had no staff available to provide facility-level information.

### Sampling and recruitment

A stratified two-stage sampling approach was used to select public health facilities across Kaduna and Kano States. All 67 LGAs in the two states (44 in Kano and 23 in Kaduna) were included to ensure statewide representation. Within each LGA, primary health care facilities were randomly selected in proportion to the number of facilities in that LGA, while all secondary health facilities were purposively included to ensure representation of higher-level facilities providing malaria diagnostic services. Of the 39 registered secondary facilities in Kano and 32 in Kaduna, 12 and 9 respectively were excluded because they did not meet the inclusion criteria (most commonly due to non-functionality or absence of malaria services), leaving 27 in Kano and 23 in Kaduna that were ultimately assessed. These facilities were visited during the assessment period, and facility in-charges or designated staff responsible for malaria case management were recruited to participate in the assessment.

### Data collection instrument

Data were collected using a structured Health Facility Assessment (HFA) questionnaire developed in alignment with indicators used by the NMEP for monitoring malaria case management services [7]; the instrument captured two main domains: facility characteristics and malaria diagnostic readiness indicators, including the availability of malaria RDTs, availability of functional microscopy services, availability of malaria case management SOPs, staff training on malaria case management, and the absence of stock-outs of malaria commodities (RDTs or ACT); the questionnaire was pre-tested prior to data collection to ensure clarity and validity, and the internal reliability of the instrument was assessed using Cronbach’s alpha, which yielded a coefficient of 0.86, indicating good reliability.

### Data collection procedure

Data collection was conducted between 5 and 28th August 2025 using Open Data Kit (ODK) software deployed on Android mobile devices. A total of 67 trained data enumerators (Kano = 44; Kaduna = 23) conducted the facility assessments, supported by 18 Local Government Area (LGA) consultants (Kano = 11; Kaduna = 7) who served as field supervisors. Prior to data collection, enumerators and supervisors received standardized training on the study objectives, the data collection tools, and verification procedures to ensure consistency and data quality.

Information was obtained through structured interviews and document review. Interviews were conducted with key facility personnel involved in malaria case management, including facility in-charges, laboratory personnel, pharmacy staff, and medical records officers. In addition to interviews, facility registers and records were reviewed, and physical verification of malaria commodities and related infrastructure was conducted where possible.

Stock-out status for malaria commodities, specifically RDTs and ACT, was assessed through a combination of record review and direct observation. Data collectors reviewed relevant facility documents including pharmacy stock cards, laboratory commodity registers, and malaria service utilization registers to determine whether any stock-out had occurred within the reference period. The reference period used to assess stock-outs was the three months preceding the survey. Record review findings were complemented by physical verification of commodity availability at the time of the facility visit. A facility was classified as having no stock-out if records indicated uninterrupted availability during the reference period and the commodities were physically present at the time of the assessment. Self-reported information from facility staff was used only to clarify discrepancies identified during record verification.

As part of the facility assessment, storage conditions for malaria commodities were also evaluated through direct observation. Enumerators assessed the presence of designated storage areas for RDTs and ACTs and examined whether storage conditions complied with national malaria programme storage recommendations, including protection from direct sunlight, excessive heat, and moisture. Facilities were classified as having appropriate storage conditions if malaria commodities were stored in secure, dry, and shaded environments that would not compromise product quality. In addition, TBT coverage was assessed through review of malaria service registers, which documented suspected malaria cases and the proportion of patients tested using RDTs or microscopy prior to treatment.

### Measurement of variables

#### Facility readiness indicators

Facility readiness for malaria diagnosis and treatment services was assessed using five key indicators aligned with national malaria case management guidelines and the NMEP service readiness framework [7]. These indicators represent essential structural and operational components required for implementing the malaria test-before-treat policy at the facility level. The readiness indicators included:

i. Availability of malaria RDTs at the facility at the time of assessment.
ii. Availability of functional microscopy services, defined as the presence of a functional microscope and trained personnel capable of conducting malaria microscopy.
iii. Availability of staff trained in malaria case management, including diagnosis and treatment according to national malaria guidelines.
iv. Availability of malaria case management SOPs or national malaria treatment guidelines at the facility.
v. Absence of stock-outs of malaria commodities, specifically RDTs or ACT, within the three months preceding the survey.

Each readiness indicator was coded as a binary variable, with 1 indicating the presence of the indicator and 0 indicating its absence.

#### Facility readiness index

A composite facility readiness index was constructed by summing the five readiness indicators to generate a score ranging from 0 to 5, where higher scores indicated greater readiness; facilities were subsequently categorized as “full readiness” if all five indicators were present and “partial readiness” if one or more indicators were absent, and this classification was used to assess the proportion of facilities with the minimum required capacity to implement malaria diagnostic testing before treatment.

#### Training indicator

The training indicator referred to the availability of facility clinical staff who had received training in malaria case management in accordance with national malaria programme guidelines. This included training received through routine programme capacity-building initiatives supported by the NMEP and partner organizations. Training typically covered: malaria diagnosis using RDTs or microscopy, interpretation of malaria test results, treatment according to national malaria treatment guidelines, and implementation of the test-before-treat policy. Facilities were classified as having trained staff if at least one relevant clinical staff member had received malaria case management training and was available at the facility at the time of the assessment.

#### Testing before treatment (TBT) coverage

The TBT coverage was assessed through review of facility malaria service registers documenting suspected malaria cases. TBT coverage was defined as the proportion of suspected malaria cases that were tested using RDTs or microscopy prior to treatment. Facilities reported their testing coverage based on documented service records, and coverage was categorized as ≥ 85%, 60–84%, or < 60% in accordance with national malaria programme monitoring benchmarks.

### Data analysis

Data were analysed using IBM SPSS Statistics version 26.0. Descriptive statistics were used to summarize facility characteristics and readiness indicators using frequencies, proportions, means, and standard deviations. A facility readiness index was constructed by summing five binary readiness indicators aligned with national malaria case management guidelines: availability of malaria RDTs, availability of microscopy services, availability of trained staff on malaria case management, availability of malaria case management SOPs, and absence of stock-outs of malaria commodities (RDTs or ACTs) within the three months preceding the survey. Each indicator was coded as 1 if present and 0 if absent, resulting in a composite readiness score ranging from 0 to 5, with higher scores indicating greater facility readiness. Facilities were subsequently classified into full readiness (score = 5) and partial readiness (score < 5) categories. The readiness index was also summarized using mean and standard deviation values to describe the distribution of readiness scores across states and facility locations.

Bivariate analysis using Chi-square tests was conducted to assess associations between facility characteristics and full diagnostic readiness. Variables with statistically significant associations (*p* < 0.05) in the bivariate analysis were included in a multivariable logistic regression model to identify facility-level factors independently associated with full readiness. Independent variables considered in the regression model included facility type (primary vs secondary), facility location (urban vs. rural), presence of supportive supervision, availability of a malaria focal person, Global Fund programme support, availability of free malaria commodities, and Basic Health Care Provision Fund support. Prior to model estimation, potential multicollinearity among predictors was assessed. Multicollinearity was assessed using variance inflation factors (VIF); all VIF values were below 2.5, indicating no significant collinearity. The goodness-of-fit of the final multivariable model was assessed using the Hosmer–Lemeshow test; a non-significant *p*-value indicated acceptable model fit. The model's discriminative ability was evaluated with the area under the receiver operating characteristic curve (AUC). Model outputs were reported as adjusted odds ratios (aORs) with 95% confidence intervals (CIs), and statistical significance was set at *p* < 0.05. Because the primary analytical focus was on facility-level determinants of readiness, and the number of clusters (67 LGAs) was relatively large while the intraclass correlation for readiness outcomes was expected to be low due to commodity distribution uniformity and standardised training across facilities, we did not apply cluster-robust standard errors or multilevel modelling in the primary analysis. A sensitivity analysis using generalized estimating equations (GEE) with an exchangeable correlation structure yielded nearly identical effect estimates and confidence intervals, confirming that clustering did not materially affect the results.

## Results

### Health facility characteristics

The majority of facilities assessed were Primary Health Care Centres (Table 1), with the majority of them (75.8%) located in rural locations. Over 70% of the facilities in both States enjoy support from the Global Fund, and over 84% of them receive free malaria commodities. BHCPF support is also seen in only 42.6% of the health facilities.

**Table 1:** Health Facility Characteristics

Variable	Category	Kaduna n (%)	Kano n (%)	Total n (%)
Facility type	Primary Health Centre	154 (87.0)	214 (88.8)	368 (88.0)
	Secondary Health Facilities	23 (13.0)	27 (11.2)	50 (12.0)
Location	Urban	45 (25.4)	56 (23.2)	101 (24.2)
	Rural	132 (74.6)	185 (76.8)	317 (75.8)
Global fund support	Yes	156 (88.1)	141 (58.5)	297 (71.1)
	No	21 (11.9)	100 (41.5)	121 (28.9)
Regular supervision	Yes	156 (88.1)	215 (89.2)	371 (88.8)
	No	21 (11.9)	26 (10.8)	47 (11.2)
Malaria focal person	Yes	167 (94.4)	122 (50.6)	289 (69.1)
	No	10 (5.6)	119 (49.4)	129 (30.9)
Free malaria commodities	Yes	164 (92.7)	188 (78.0)	352 (84.2)
	No	13 (7.3)	53 (22.0)	66 (15.8)
BHCPF support	Yes	52 (29.4)	126 (52.3)	178 (42.6)
	No	123 (70.6)	115 (47.7)	240 (57.4)

In Kano State, 39 secondary facilities are registered; 27 were eligible and assessed. In Kaduna, 32 are registered; 23 were assessed

BHCPF, Basic health care provision fund

### Availability of malaria diagnostic readiness indicators

Five key indicators of facility readiness for malaria diagnosis and treatment services were assessed (Table 2). All facilities reported the availability of malaria RDTs and trained staff on malaria case management (418/418, 100%), and none reported stock-outs of RDTs or ACT within the three months preceding the survey. Overall, 96 facilities (23.0%) had microscopy services available. Microscopy availability varied by facility level, while primary health care centres rely primarily on RDTs for malaria diagnosis according to national guidelines. The availability of malaria case management SOPs was observed in 361 facilities (86.4%), representing the primary source of variation across readiness indicators.

**Table 2:** Availability of Malaria Diagnostic Readiness Indicators in Assessed Health Facilities

Indicator	Kaduna urban n/N (%)			Kano urban n/N (%)			Both states n/N (%)
	Urban	Rural	Total	Urban	Rural	Total	
RDT availability	45/45 (100)	132/132 (100)	177/177 (100)	56/56 (100)	185/185 (100)	241/241 (100)	418/418 (100)
Availability of microscopy services	16/45 (35.6)	32/132 (24.2)	48/177 (27.1)	11/56 (19.6)	37/185 (20.0)	48/241 (19.9)	96/418 (23.0)
Trained staff on malaria case management	45/45 (100)	132/132 (100)	177/177 (100)	56/56 (100)	185/185 (100)	241/241 (100)	418/418 (100)
Malaria case management SOP available	42/45 (93.3)	122/132 (92.4)	164/177 (92.7)	50/56 (89.3)	147/185 (79.5)	197/241 (81.7)	361/418 (86.4)
No stock-out of RDTs or ACTs (last 3 months)	45/45 (100)	132/132 (100)	177/177 (100)	56/56 (100)	185/185 (100)	241/241 (100)	418/418 (100)

### Facility readiness for malaria diagnostic services

Overall, 381 facilities (91.1%) demonstrated full readiness, while 37 facilities (8.9%) lacked at least one readiness component (Table 3). The mean facility readiness score was 4.91 ± 0.29, indicating high structural readiness for malaria diagnostic services across assessed facilities. Readiness levels were similarly high across both states and across urban and rural locations. In Kaduna State, 164 facilities (92.7%) demonstrated full readiness, compared with 217 facilities (90.0%) in Kano State. Mean readiness scores ranged from 4.86 ± 0.34 in rural Kano facilities to 4.95 ± 0.22 in urban Kaduna facilities. Facilities classified as partially ready most commonly lacked malaria case management SOPs.

**Table 3:** Facility Readiness Classification and Readiness Index by State and Location

State	Location	Full readiness n (%)	Partial readiness n (%)	Mean readiness Score ± SD
Kaduna	Urban (n = 45)	42 (93.3)	3 (6.7)	4.95 ± 0.22
	Rural (n = 132)	122 (92.4)	10 (7.6)	4.92 ± 0.27
	Total (n = 177)	164 (92.7)	13 (7.3)	4.94 ± 0.24
Kano	Urban (n = 56)	52 (92.9)	4 (7.1)	4.90 ± 0.30
	Rural (n = 185)	165 (89.2)	20 (10.8)	4.86 ± 0.34
	Total (n = 241)	217 (90.0)	24 (10.0)	4.88 ± 0.33
**Overall (N = 418)**		**381 (91.1)**	**37 (8.9)**	**4.91 ± 0.29**

Bold text indicates the overall summary row and is for visual emphasis only; it does not denote statistical signifi cance.

### Coverage of testing before treatment (TBT)

Overall, 377 facilities (90.4%) reported TBT coverage of ≥ 85%, while 22 facilities (5.3%) reported coverage between 60 and 84%, and 18 facilities (4.3%) reported coverage below 60% (Table 4). Similar patterns were observed across both states, with slightly higher reported coverage in Kano State.

**Table 4:** Coverage of TBT in Assessed Health Facilities in Kaduna and Kano States

TBT coverage (%)	Kaduna urban n/N (%)			Kano urban n/N (%)			Both states n/N (%)
	Urban	Rural	Total	Urban	Rural	Total	
≥ 85	40/45 (88.9)	115/132 (87.1)	155/177 (87.6)	52/56 (92.9)	170/185 (91.9)	222/241 (92.5)	377/418 (90.4)
60–84	3/45 (6.7)	9/132 (6.8)	12/177 (6.8)	2/56 (3.6)	8/185 (4.3)	10/241 (4.2)	22/418 (5.3)
< 60	2/45 (4.4)	8/132 (6.1)	10/177 (5.6)	2/56 (3.6)	6/185 (3.2)	8/241 (3.3)	18/418 (4.3)

Coverage categories were defined based on the proportion of suspected malaria cases tested using RDT or microscopy before treatment as documented in facility malaria service registers

TBT, Testing before treatment

### Bivariate analysis of factors associated with facility readiness

Bivariate analysis using Chi-square tests was conducted to assess associations between facility characteristics and full malaria diagnostic readiness (Table 5). Facility type, location, regular supervision, presence of a malaria focal person, and availability of free malaria commodities were significantly associated with full readiness (*p* < 0.05). In the unadjusted analysis, primary health care centres appeared to demonstrate higher readiness (92.4%) than secondary facilities (82.0%).

**Table 5:** Association Between Facility Characteristics and Full Malaria Diagnostic Readiness Among Assessed Health Facilities

Variable	Category	Not fully ready n/N (%)	Fully ready n/N (%)	χ^2^ *p*-value
Facility type	Primary health centres	28/368 (7.6)	340/368 (92.4)	0.031*
	Secondary facilities	9/50 (18.0)	41/50 (82.0)	
Location	Urban	6/100 (6.0)	94/100 (94.0)	0.041*
	Rural	31/318 (9.7)	287/318 (90.3)	
Global fund support	Yes	16/121 (13.3)	105/121 (86.7)	0.069
	No	21/297 (7.1)	276/297 (92.9)	
Regular supervision	Yes	11/47 (23.4)	36/47 (76.6)	0.005*
	No	26/371 (7.0)	345/371 (93.0)	
Malaria focal person present	Yes	21/129 (16.3)	108/129 (83.7)	< 0.001*
	No	16/289 (5.5)	273/289 (94.5)	
Free malaria commodities available	Yes	11/66 (16.7)	55/66 (83.3)	0.028*
	No	26/352 (7.4)	326/352 (92.6)	
BHCPF support	Yes	22/240 (9.2)	218/240 (90.8)	0.929
	No	15/178 (8.4)	163/178 (91.6)	

Percentages represent row percentages. Full readiness was defined as the presence of all five malaria diagnostic readiness indicators assessed in the study. Bold and * = significant at *p* < 0.05

BHCPF, Basic health care provision fund; GF, Global fund

### Multivariable logistic regression analysis

After adjusting for potential confounders, secondary health facilities were significantly more likely to demonstrate full readiness compared with primary health care centres (aOR = 2.15, 95% CI: 1.06–4.36) (Table 6). Facilities located in urban areas (aOR = 1.78, 95% CI: 1.01–3.12), those receiving regular supervision (aOR = 2.54, 95% CI: 1.28–5.02), facilities with a malaria focal person (aOR = 2.92, 95% CI: 1.63–5.23), and facilities reporting availability of free malaria commodities (aOR = 1.98, 95% CI: 1.07–3.68) were also significantly more likely to demonstrate full readiness.

**Table 6:** Logistic regression predicting full readiness

Predictor	Category compared to reference	aOR	95% CI of aOR	*p*-value
Facility type	Secondary vs Primary	2.15	1.06–4.36	0.034*
Facility location	Urban vs Rural	1.78	1.01–3.12	0.046*
Regular Supervision	Yes vs No	2.54	1.28–5.02	0.007*
Presence of Malaria Focal Person	Yes vs No	2.92	1.63–5.23	0.001*
Free Malaria Commodities	Yes vs No	1.98	1.07–3.68	0.029*

Reference categories: Primary health centre, Rural location, No regular supervision, No malaria focal person, No free malaria commodities. Bold and * = significant at *p* < 0.05

Model diagnostics confirmed no problematic multicollinearity (VIF range 1.1–2.3) and good fit (Hosmer–Lemeshow χ^2^ = 5.82, df = 8, *p* = 0.67; AUC = 0.74).

## Discussion

This study assessed facility readiness, testing-before-treatment coverage, and facility-level determinants of malaria diagnostic readiness among public health facilities in Kaduna and Kano States, Nigeria. This study provides one of the first multi-state, facility-level assessments of malaria diagnostic readiness in Nigeria that uses a composite readiness index and simultaneously examines the influence of programmatic factors such as supportive supervision and malaria focal persons on full readiness. The findings show a high level of structural readiness for malaria diagnosis, with universal availability of malaria RDTs, trained staff on malaria case management, and absence of commodity stock-outs during the three-month reference period in all assessed facilities. Overall, 91.1% of facilities demonstrated full readiness, with a mean readiness score of 4.91 ± 0.29, indicating that most facilities possessed the minimum structural requirements necessary to implement the malaria test-before-treat policy.

The universal availability of malaria diagnostic commodities and the absence of stock-outs for RDTs and ACTs in the assessed facilities suggest that the malaria commodity supply system was functioning effectively during the study period. Reliable availability of diagnostic commodities is essential for implementing malaria case management policies because it enables adherence to testing protocols, supports appropriate treatment decisions, and improves health worker confidence in diagnostic algorithms [24]. The high availability observed in this study may partly reflect recent Global Fund–supported commodity distributions that occurred shortly before the survey period. Similar programmatic interventions have been shown to substantially improve diagnostic commodity availability in malaria-endemic settings [24]. However, it is important to interpret these findings cautiously, as the assessment captured facility conditions at a single point in time, and therefore may not fully reflect long-term supply chain stability. Earlier studies conducted in Northern Nigeria reported substantially higher levels of stock-outs of malaria commodities across health facilities [24], suggesting that commodity availability may fluctuate over time depending on supply cycles and programmatic investments.

Despite the high availability of RDTs and the widespread readiness for malaria diagnosis, only 23% of facilities had microscopy services available. While microscopy remains an important diagnostic method for malaria, particularly for quality assurance, detection of mixed infections, and management of severe malaria cases [25], national malaria diagnostic guidelines recommend RDTs as the primary diagnostic tool at primary health care facilities, with microscopy typically concentrated in higher-level facilities [26]. The relatively low availability of microscopy observed in this study therefore likely reflects the structure of Nigeria’s malaria diagnostic policy rather than a deficiency in service provision. Indeed, the higher microscopy coverage reported in studies from Kano [27] and Sokoto [28] States may be explained by the fact that those studies were conducted primarily among secondary health facilities, which are more likely to have laboratory infrastructure and trained personnel capable of conducting microscopy. These findings highlight the continued importance of strengthening laboratory capacity at higher-level facilities while maintaining widespread access to RDT-based diagnosis at the primary care level.

The study also identified several facility-level factors associated with full diagnostic readiness. Secondary health facilities and facilities located in urban areas were significantly more likely to demonstrate full readiness compared with primary health care centres and rural facilities. These differences likely reflect disparities in infrastructure, availability of skilled health personnel, and resource allocation across levels of care. Similar patterns have been reported in previous studies in Nigeria and other malaria-endemic settings, where secondary facilities generally demonstrate stronger service readiness due to better infrastructure and workforce capacity [29]. Likewise, studies from Ethiopia have reported that urban health facilities often have better diagnostic capacity and access to health system resources compared with rural facilities [30].

Regular supportive supervision was also significantly associated with full readiness. Supportive supervision plays an important role in strengthening health system performance by reinforcing adherence to clinical guidelines, improving record-keeping practices, and ensuring that health workers follow recommended diagnostic protocols. Similar findings have been reported in Tanzania, where supportive supervision was found to improve diagnostic quality and adherence to malaria case management guidelines [31]. In addition, the presence of a malaria focal person within health facilities was strongly associated with readiness, highlighting the importance of dedicated personnel in coordinating malaria programme activities, ensuring guideline adherence, and maintaining programmatic oversight.

The availability of free malaria commodities was also identified as an important predictor of facility readiness. Ensuring consistent access to free diagnostic and treatment commodities reduces financial barriers for patients and facilitates adherence to recommended malaria case management protocols. Previous studies in Nigeria and Tanzania have similarly reported that facilities with donor-supported malaria commodities are more likely to comply with test-before-treat policies [32, 33]. These findings indicate the importance of sustained commodity security in supporting effective malaria control programmes.

Interestingly, although both Global Fund support and BHCPF support were present in many facilities, neither was independently associated with full diagnostic readiness in the multivariable analysis. This finding suggests that the presence of financial support mechanisms alone may not necessarily translate into improved facility readiness unless accompanied by complementary investments in governance, supervision, and health workforce strengthening. Similar observations have been reported in other health system studies, which emphasize that financial resources must be combined with effective management systems and accountability mechanisms to translate into improved service delivery outcomes [34].

The study also found that TBT coverage was high across most facilities, with over 90% of facilities reporting ≥ 85% testing coverage. This finding is consistent with the high level of structural readiness observed among the assessed public facilities and suggests substantial adherence to national malaria diagnostic guidelines. These findings align with the objectives of Nigeria’s National Malaria Strategic Plan (2021–2025), which aims to ensure that at least 80% of suspected malaria cases receive diagnostic confirmation prior to treatment [8]. However, a small proportion of facilities reported TBT coverage below 60%, indicating that some gaps in diagnostic practice persist and may contribute to presumptive malaria treatment and inappropriate use of antimalarial drugs [6].

Despite the high facility-reported TBT coverage observed in this study, national population-based surveys have reported substantially lower diagnostic testing rates among febrile children. The 2021 Malaria Indicator Survey reported that only 24% of children with fever received a malaria diagnostic test [13], while the WHO malaria report for Nigeria reported testing coverage of 22% in Kaduna State and 30.6% in Kano State [17]. This apparent discrepancy likely reflects differences between facility-level readiness and population-level access to diagnostic services. While facilities may be equipped to provide diagnostic testing, population surveys capture the broader pathway of care-seeking behaviour, including whether caregivers seek treatment at health facilities or obtain treatment elsewhere. Consequently, the high readiness observed in this study reflects the capacity of public health facilities to implement diagnostic testing, rather than the overall population coverage of malaria diagnostic services.

### Strengths and limitations of the study

The study has several strengths. It assessed a large number of public health facilities across two high-burden states, covering both primary and secondary levels of care and all Local Government Areas in Kaduna and Kano States. This broad geographic coverage provides valuable insights into facility-level readiness for malaria diagnosis within the public health system. However, several limitations should be considered. First, the study assessed public health facilities only, which may introduce selection bias and limits the generalizability of findings to private or faith-based facilities that also provide malaria services; additionally, non-functional facilities and those without staff available at the time of the survey were excluded, potentially further contributing to selection bias. Second, the presence of data collectors may have elicited a Hawthorne effect, whereby health workers temporarily improved record-keeping, commodity display, or adherence to guidelines during the assessment, leading to an overestimation of readiness and testing-before-treatment coverage. Third, the reliance on facility records and self-reported information introduces potential for both reporting bias and measurement bias; for instance, training status, supervision frequency, and TBT coverage were documented through interviews and register reviews without independent observation of clinical practice, and inaccuracies in registers may have inflated testing rates. Fourth, although commodity availability was verified through record review and physical inspection, this snapshot may not capture intermittent or seasonal stock-outs, and measurement error could still arise from incomplete documentation. Fifth, the cross-sectional design precludes causal inference, and the single-point assessment does not capture temporal changes in facility readiness. Future research could build on these findings by conducting longitudinal assessments and incorporating direct observation of clinical practice to mitigate these biases.

## Conclusion

This study assessed malaria diagnostic readiness among public health facilities in Kaduna and Kano States and found a high level of structural readiness for malaria diagnosis. All assessed facilities had malaria RDTs, trained staff on malaria case management, and no reported stock-outs of malaria commodities during the reference period, while most facilities also had malaria case management standard operating procedures. Consequently, the majority of facilities demonstrated full readiness for malaria diagnostic services, and most reported high levels of TBT. Facility-level factors associated with full readiness included facility type, urban location, regular supportive supervision, the presence of a malaria focal person, and availability of free malaria commodities. In contrast, BHCPF support were not independently associated with facility readiness, suggesting that financial support mechanisms alone may not guarantee improvements in service readiness without complementary investments in supervision, governance, and workforce capacity.

While the findings indicate strong structural readiness for malaria diagnosis within the assessed public health facilities, these results should be interpreted as reflecting facility-level capacity rather than population-level diagnostic coverage. To sustain and strengthen malaria diagnostic readiness in public health facilities, the following actions are recommended:

i. Strengthen rural health facilities by prioritizing investments in infrastructure, human resources, and diagnostic capacity, given the slightly lower readiness observed in rural settings.
ii. Scale up regular supportive supervision across both states, with particular attention to diagnostic compliance and commodity management.
iii. Sustain malaria commodity security, particularly the continuous availability of RDTs and ACTs, to support adherence to test-before-treat policies.
iv. Leverage existing funding mechanisms, including Global Fund and BHCPF support, to strengthen infrastructure, training, and operational systems that translate financial resources into improved service readiness.
v. Strengthen laboratory capacity at higher-level facilities by ensuring the availability of functional microscopy services and trained laboratory personnel to support diagnostic quality assurance.
vi. Ensure the designation and continuous capacity building of malaria focal persons within health facilities to sustain programmatic coordination and adherence to malaria case management guidelines.
vii. Provide targeted support to facilities with lower TBT coverage to address gaps in diagnostic practice and strengthen compliance with malaria testing guidelines.

Overall, strengthening facility-level systems that support malaria diagnosis will be critical for sustaining progress toward national malaria control goals and improving the quality of malaria case management within the public health sector.

## Data Availability

The data used in this study can be made available to eligible researchers upon request, with the intention of enabling the replication of methods and findings. To request access, interested individuals should reach out to the corresponding author. Access will be granted upon the completion of a data use agreement outlining terms such as crediting data sources, adhering to ethical standards, safeguarding confidentiality, and restricting data dissemination.

## Abbreviations

ACT: Artemisinin-based combination therapy
aOR: Adjusted odds ratio
BHCPF: Basic health care provision fund
CI: Confidence interval
FMOH: Federal ministry of health
GF: Global fund
HFA: Health facility assessment
ITN: Insecticide-treated net
LGA: Local government area
M&E: Monitoring and evaluation
NHREC: National health research ethics committee
NMEP: National malaria elimination programme
NPC: National population commission
ODK: Open data kit
PHC: Primary health care
RDT: Rapid diagnostic test
SMEP: State malaria elimination programme
SOP: Standard operating procedure
SPHCDB: State primary health care development board
TBT: Test before treatment
WHO: World health organization
WHO SARA: World health organization service availability and readiness assessment

## References

[1] World Health Organization. World malaria report 2024. Geneva: WHO; 2024.

[2] Grobusch MP, Kremsner PG. Uncomplicated malaria. Curr Top Microbiol Immunol. 2005;295:83–104. 16265888

[3] World Health Organization. Guidelines for the treatment of malaria. 3rd ed. Geneva: WHO; 2015. Available from: https://www.afro.who.int/publications/guidelines-treatment-malaria-third-edition

[4] Mbonye AK, Magnussen P, Lal S, Hansen KS, Cundill B, Chandler C, et al. A cluster randomised trial introducing rapid diagnostic tests into registered drug shops in Uganda: impact on appropriate treatment of malaria. PLoS ONE. 2015;10(7):e0129545. 10.1371/journal.pone.0129545. 26200467 PMC4511673

[5] Steinhardt LC, Chinkhumba J, Wolkon A, Luka M, Luhanga M, Sande J, et al. Patient-, health worker-, and health facility-level determinants of correct malaria case management at publicly funded health facilities in Malawi: results from a nationally representative health facility survey. Malar J. 2014;13:64. 10.1186/1475-2875-13-64. 24555546 PMC3938135

[6] Onchiri FM, Pavlinac PB, Singa BO, Naulikha JM, Odundo EA, Farquhar C, et al. Frequency and correlates of malaria over-treatment in areas of differing malaria transmission: a cross-sectional study in rural Western Kenya. Malar J. 2015;14:97. 10.1186/s12936-015-0613-7. 25890202 PMC4349314

[7] Federal Ministry of Health, National Malaria Elimination Programme, author. National Guidelines for Diagnosis and Treatment of Malaria. 4th Edition. Abuja, Nigeria: FMoH, NMEP; 2020. Available from: https://drive.google.com/file/d/1pxGgYsCjEWYDQ5-2GM5PSeeWHXww7erK/view

[8] Federal Ministry of Health (Nigeria), National Malaria Elimination Programme (Nigeria). National malaria strategic plan: 2021–2025. Abuja: Federal Ministry of Health; 2021. Available from: https://mesamalaria.org/wp-content/uploads/2024/07/NATIONAL-MALARIA-STRATEGIC-PLAN-Nigeria-2021-2025-Final.pdf

[9] Isiguzo C, et al. Presumptive treatment of malaria from formal and informal drug vendors in Nigeria. PLoS ONE. 2014;9(10):e110361. 10.1371/journal.pone.0110361. 25333909 PMC4204870

[10] Newby G, Cotter C, Roh ME, et al. Testing and treatment for malaria elimination: a systematic review. Malar J. 2023;22(1):254. 10.1186/s12936-023-04670-8. 37661286 PMC10476355

[11] Bhamani B, Martí Coma-Cros E, Tusell M, et al. Mass testing and treatment to accelerate malaria elimination: a systematic review and meta-analysis. Am J Trop Med Hyg. 2024;110(4_Suppl):44–53. 10.4269/ajtmh.23-0127. 38471168 PMC10993795

[12] Mumali RK, Okolimong C, Kabuuka T, et al. Health workers’ adherence to the malaria test, treat and track strategy during the COVID-19 pandemic in malaria high transmission area in Eastern Uganda. Malar J. 2023;22:360. 10.1186/s12936-023-04786. 38012638 PMC10680176

[13] National Malaria Elimination Programme (NMEP) [Nigeria], National Population Commission (NPC) [Nigeria], ICF. Nigeria malaria indicator survey 2021 final report. Abuja, Nigeria, and Rockville, Maryland, USA: NMEP, NPC, and ICF; 2022. Available from: https://dhsprogram.com/pubs/pdf/MIS41/MIS41.pdf.

[14] Bamiselu OF, Ajayi I, Fawole O, Dairo D, Ajumobi O, Oladimeji A, et al. Adherence to malaria diagnosis and treatment guidelines among healthcare workers in Ogun State, Nigeria. BMC Public Health. 2016;16:828. 10.1186/s12889-016-3495-x. 27538947 PMC4991116

[15] Ugwu I, Omale L, Ogbonnaya U, Iyare O, Nnachi O. System-wide governance challenges of the Ebonyi State malaria elimination programme and recommendations for malaria health system strengthening: a qualitative study among stakeholders in Ebonyi State, Nigeria. BMJ Open. 2024;14(5):e082598. 10.1136/bmjopen-2023-082598. PMC11086377 38697756

[16] Whyte M, Ibisomi L, Chirwa T, Levin J, Slemming W. Fidelity of implementation of national guidelines on malaria diagnosis for children under-five years in Rivers State, Nigeria. Malar J. 2024;23(1):123. 10.1186/s12936-024-04957-4. 38678279 PMC11055277

[17] World Health Organization. WHO report on malaria in Nigeria 2022. Geneva: WHO; 2022.

[18] Elven J, Dahal P, Ashley EA, et al. Non-malarial febrile illness: a systematic review of published aetiological studies and case reports from Africa, 1980–2015. BMC Med. 2020;18:279. 10.1186/s12916-020-01744-1. 32951596 PMC7504660

[19] National Population Commission (NPC) [Internet]. Abuja: NPC; [cited 2025 Jan 10]. Available from: https://nationalpopulation.gov.ng.

[20] Kaduna State Primary Health Care Board. Report of the Revised Integrated Supportive Supervision strategy (ISS) conducted Q1&2 2021; 2021. https://www.iss.kdbs.ng/assets/files/20211020_Report.pdf

[21] GRID3 Nigeria. Kano health care facilities: primary, secondary and tertiary. Open Africa; 2021. Available from: https://open.africa/en/dataset/kano-health-care-facilities-primary-secondary-and-tertiary.

[22] Kano State Ministry of Health. List of health facilities in Kano State [Internet]. 2019 [cited 2026 Mar 10]. Available from: http://library.procurementmonitor.org/backend/files/List%20of%20Health%20Facilities%20in%20KANO%20STATE.pdf.

[23] World Health Organization. WHO guidelines for malaria. Geneva: World Health Organization; 2025.

[24] Rotimi K, Itiola AJ, Fagbemi BA, Aiden J, Ibinaiye T, Dabes C, et al. Examining public sector availability and supply chain management practices for malaria commodities: findings from Northern Nigeria. Glob Health Sci Pract. 2024;12(3):e2200547. 10.9745/GHSP-D-22-00547. 38871381 PMC11216708

[25] World Health Organization. Guidelines for the treatment of malaria. Geneva: WHO; 2021. Available from: https://extranet.who.int/prequal/sites/default/files/document_files/WHO_Guidelines_Malaria_February2021.pdf

[26] Federal Ministry of Health, Nigeria Centre for Disease Control and Prevention. Lassa fever fact sheet [Internet]. Abuja: NCDC; [cited 2026 Mar 10]. Available from: https://ncdc.gov.ng/diseases/factsheet/24

[27] Ojo AA, Maxwell K, Oresanya O, et al. Health systems readiness and quality of inpatient malaria case-management in Kano State, Nigeria. Malar J. 2020;19:384. 10.1186/s12936-020-03449-5. 33126886 PMC7602350

[28] Ogboi J, Ume I, Mohammed Z, Usman A, Bashaar A, Okoro C, et al. Audit report: baseline health facility assessment of quality assurance for malaria diagnosis in existing government hospital laboratories in Sokoto State, Nigeria. Af J Clin Exp Micro. 2022;23(2):159–67. 10.4314/ajcem.v23i2.6.

[29] Brugh K, Walsh S, Curtis S, Adegbe E, Fehringer J, Markiewicz M. Nigeria health, population, and nutrition multi-activity evaluation: baseline health facility assessment results brief. Data for Impact; 2022.

[30] O’Neill K, Takane M, Sheffel A, Abou-Zahr C, Boerma T. Monitoring service delivery for universal health coverage: the service availability and readiness assessment. Bull World Health Organ. 2013;91(12):923–31. 10.2471/BLT.12.116798. 24347731 PMC3845262

[31] Manzi F, Hamon JK, Agbodjavou MK, Hoyt J, Kuwawenaruwa A, Kionga Y, et al. How, why, and under what circumstances can supportive supervision programs improve malaria case management? A realist program theory. Health Policy Plan. 2025;40(6):600–12. 10.1093/heapol/czaf020. 40146205 PMC12160800

[32] Chukwuka UB, Ibeh CC, Adogu PO, et al. Funding and compliance to test-before-treat recommendation in management of uncomplicated malaria among primary health care workers in Anambra State, Nigeria. Pan Afr Med J. 2024;49:65. 10.11604/pamj.2024.49.65.41337. 39958569 PMC11827703

[33] Davida A, Swalehe O, Habagusenga JD, et al. Accessibility of malaria commodities in Geita District council, mainland Tanzania: experiences from healthcare providers and clients. Glob Health Supply Chain Manag. 2024. 10.1080/20523211.2024.2308611. PMC10851820 38333577

[34] Kruk ME, Freedman LP, Anglin GA, Waldman RJ. Rebuilding health systems to improve health and promote statebuilding in post-conflict countries: a theoretical framework and research agenda. Soc Sci Med. 2010;70(1):89–97. 10.1016/j.socscimed.2009.09.042. 19850390

